# Cerebral salt wasting after traumatic brain injury: a review of the literature

**DOI:** 10.1186/s13049-015-0180-5

**Published:** 2015-11-11

**Authors:** Jan Leonard, Raymond E. Garrett, Kristin Salottolo, Denetta S. Slone, Charles W. Mains, Matthew M. Carrick, David Bar-Or

**Affiliations:** Department of Trauma Research, Swedish Medical Center, 501 E. Hampden Ave, Englewood, CO 80113 USA; Department of Trauma Research, St. Anthony Hospital, 11600 W. 2nd Place, Lakewood, CO 80228 USA; Department of Trauma Research, The Medical Center of Plano, 3901 West 15th St, Plano, TX 75075 USA; Craig Hospital, 3425 S. Clarkson St, Englewood, CO 80113 USA; Trauma Services Department, Swedish Medical Center, 501 E. Hampden Ave, Englewood, CO 80113 USA; Trauma Services Department, St. Anthony Hospital, 11600 W. 2nd Place, Lakewood, CO 80228 USA; Trauma Services Department, The Medical Center of Plano, 3901 West 15th St, Plano, TX 75075 USA

**Keywords:** Hyponatremia, Water-electrolyte imbalance, Traumatic brain injury, Incidence, Natriuretic peptides, Review

## Abstract

Electrolyte imbalances are common among patients with traumatic brain injury (TBI). Cerebral salt wasting (CSW) is an electrolyte imbalance characterized by hyponatremia and hypovolemia. Differentiating the syndrome of inappropriate antidiuretic hormone and CSW remains difficult and the pathophysiological mechanisms underlying CSW are unclear. Our intent was to review the literature on CSW within the TBI population, in order to report the incidence and timing of CSW after TBI, examine outcomes, and summarize the biochemical changes in patients who developed CSW. We searched MEDLINE through 2014, hand-reviewed citations, and searched abstracts from the American Association for the Surgery of Trauma (2003–2014). Publications were included if they were conducted within a TBI population, presented original data, and diagnosed CSW. Publications were excluded if they were review articles, discussed hyponatremia but did not differentiate the etiology causing hyponatremia, or presented cases with chronic disease. Fifteen of the 47 publications reviewed met the selection criteria; nine (60 %) were case reports, five (33 %) were prospective and 1 (7 %) was a retrospective study. Incidence of CSW varied between 0.8 - 34.6 %. The populations studied were heterogeneous and the criteria used to define hyponatremia and CSW varied. Though believed to play a role in the development of CSW, increased levels of natriuretic peptides in patients diagnosed with CSW were not consistently reported. These findings reinforce the elusiveness of the CSW diagnosis and the need for strict and consistent diagnostic criteria.

**Electronic supplementary material:**

The online version of this article (doi:10.1186/s13049-015-0180-5) contains supplementary material, which is available to authorized users.

## Introduction

Brain injury or illness, including traumatic brain injury (TBI), is frequently associated with perturbations in water balance including hyponatremia [1]. When hyponatremia accompanies acute brain pathology, the differential diagnosis includes fluid volume depletion, effects of certain medications, syndrome of inappropriate antidiuretic hormone (SIADH), and salt wasting syndromes, in particular cerebral salt wasting (CSW).

First described by Peters et al. in 1950 [2], CSW is characterized by a renal loss of sodium following intracranial disorders, resulting in hyponatremia and hypovolemia [1, 3–5]. CSW is complex and poorly understood; it can easily be confused with SIADH and the aforementioned differential diagnoses. A clinician’s ability to promptly distinguish CSW is crucial; the emergent therapy for all of these conditions is similar (hypertonic saline and/or 0.9 % saline), but sub-acute therapy with vaptans, a class of drugs that antagonize vasopressin receptors, is contraindicated in salt wasting states [6]. Additionally, hypovolemia in CSW necessitates replacement of sodium and water, whereas the combination of hyponatremia and excess fluid in SIADH is treated via water restriction. If left untreated, severe hyponatremia can result in seizures and worsening cerebral edema [7]. When hyponatremia is suboptimally treated, the patient is at an increased risk of delayed ischemic deficits and/or osmotic demyelination leading to disability and excess mortality [8].

The mechanisms leading to CSW have yet to be clearly defined and no single etiology is believed to be the sole cause of CSW. It is hypothesized that CSW develops because of increased levels of natriuretic peptides and changes in the sympathetic nervous system, the renin-angiotensin-aldosterone system, and adrenomedullin [7].

CSW is most frequently studied in patients with aneurysmal subarachnoid hemorrhage (aSAH) [5]. This population experiences a high incidence of hyponatremia, observed in up to 57 % of patients [9]. Although TBI is also associated with hyponatremia, CSW has rarely been studied in the TBI population, and much of the literature consists of case reports, reviews and small cohort studies that focus on incidence, etiology, and biochemical changes. Little information is available on outcomes in patients with CSW after TBI.

Our objective was to review the literature on CSW within the TBI population in order to define the incidence and timing of CSW after TBI, examine outcomes, and summarize the biochemical changes in patients who developed CSW.

## Review

### Methods

We performed a methodical search of the CSW medical literature through 2014 using the online database MEDLINE. Initial search criteria phrases included “cerebral salt wasting” AND “traumatic brain injury” OR “head trauma”; the search was limited to English-language publications. Citations from published articles were hand-reviewed and we searched abstracts from the American Association for the Surgery of Trauma (AAST) using the AAST online collection of past abstracts (2003–2014).

Studies were included in this review if they: 1) were conducted within patients who suffered a TBI, 2) presented original data by way of case reports, prospective and retrospective observational studies, or randomized controlled clinical trials, and 3) diagnosed cerebral salt wasting. Publications were excluded if they were review articles, discussed hyponatremia but did not differentiate the etiology causing hyponatremia, or were case reports in patients with chronic disease.

The Preferred Reporting Items for Systematic Reviews and Meta-Analyses (PRISMA) Statement [10] checklist was used to ensure appropriate collection of variables. For this review we collected and tabulated information on publication year and source, study design, study size, patient inclusion and exclusion criteria, patient demographics, cause of TBI, results of head computed tomography (CT) scans, and patient outcomes. We recorded levels of atrial natriuretic peptide (ANP), brain natriuretic peptide (BNP), antidiuretic hormone (ADH), aldosterone, cortisol, adrenocorticotropic hormone (ACTH), and plasma renin activity in order to summarize biochemical changes that are potentially part of the pathophysiological pathway leading to CSW.

## Results

### Study identification and selection

**Fig. 1:**
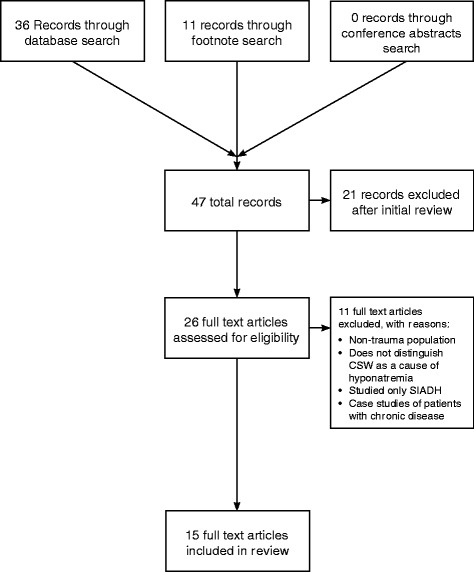
PRISMA flow chart demonstrating study identification and selection process

A total of 36 unique articles were initially identified through electronic database search, 0 through a search of conference abstracts, and 11 through hand searching citations, resulting in 47 total publications (Fig. 1). The initial review eliminated 21 publications that were either review articles or were clearly not conducted in TBI patients. Twenty-six full text articles were then assessed for eligibility and eleven additional publications were excluded for not meeting study selection criteria.

Fifteen of 47 (32 %) publications remained in our review (Additional files 1 and 2). Nine (60 %) of the publications were case reports that presented a total of 10 cases, five (33 %) were prospective and 1 (7 %) was a retrospective study. Seven of the fifteen (47 %) articles were published in a neurology or neurosurgery journal [11–17], six (40 %) were in a pediatric journal [17–22] and the remainder were published within journals of varied specialties [23–25].

### Patient inclusion

Patient population was inconsistently defined across cohort studies (Additional files 1 and 2). Five of the 9 case reports (56 %) were from American institutions [16, 17, 21, 22, 25]. The remaining case reports and cohort studies were conducted worldwide. Studies were published over a 24 year span (1988–2011). Of the six cohort studies, one study was conducted in pediatric patients [18], one study included patients aged ≥20 years [11], and the remaining four studies did not mention specific age requirements and included patients ranging from 0 – 92 years old [12–14, 23]. Eight of the 10 presented cases were of patients under the age of 18 [17, 19–22, 24, 25]. One study examined a population of severely injured patients, defined as those with brain injury and a Glasgow Coma Score (GCS) less than 9 [12]. Two studies excluded spinal cord injuries [11, 13] and three studies excluded concomitant chronic conditions such as renal, thyroid, and adrenal disease, hypertension, and cardiopathy [11, 12, 23].

While all patients suffered a TBI, the particular type varied (Additional files 1 and 2). Among patients with any hyponatremia, Lohani et al. observed thirty-three percent of patients had more than one lesion; intraparenchymal lesions were most frequent (89 %), followed by epidural lesions (33 %), pneumocephalus (22 %), and traumatic subarachnoid hemorrhage (tSAH, 11 %) [11]. Moro et al. found patients with any hyponatremia experienced cerebral contusions (46 %), chronic subdural hematomas (28 %), acute subdural hematomas (16 %), acute epidural hematomas (8 %), and diffuse axonal injuries (1 %) [13].

### Incidence of cerebral salt wasting

The incidence of CSW after TBI ranged from 0.8 to 34.6% (Additional file 1) and was highest in the study restricted to patients with a GCS less than nine [12]. Unfortunately, CSW was not consistently or always concretely defined. Diagnoses of CSW in three of the cohort studies were made when the following criteria were observed: 1) hyponatremia (<135 mmol/L), increase in urine sodium concentration (>18 mmol/L), large urine volume (>3000 mL/d), and low blood volume [23], 2) urine osmolality/plasma osmolality >1, reduced plasma osmolality (<270 mmol/kg H_2_O) and natremia (<137 mmol/l) combined with polyuria (>150 % of fluid intake) and clinical and biochemical signs of reduced extracellular volume [18], and 3) serum sodium level <136 mEq/L that required additional sodium retention therapy and massive sodium excretion with negative sodium and water in-out balances [13]. The remaining three cohort studies used varying sodium levels as a start – <130 mEq/L [11], ≤ 130 mEq/l [12], and <135 mmol/l [14] – and then evaluated clinical data, though did not report pre-defined values that would qualify as CSW. Due to varying study inclusion and CSW diagnostic criteria, we did not perform a pooled data analysis.

### Timing from traumatic brain injury to development of cerebral salt wasting

The timing from injury to the development of CSW appears to vary. Lohani et al. found hyponatremia occurred late in the first week and early in the second week after injury [11], whereas Moro et al. reported that most patients presented with hyponatremia within 3 days of injury [13]. Neither of these cohort studies indicated when surgery was performed relevant to development of hyponatremia [26] or specifically reported the time from injury to the development of CSW. Eight pediatric and 2 adult cases of CSW were presented (Additional file 2). In the pediatric patients, three developed CSW 2 days after injury [17, 22, 25], two developed CSW approximately 1 week after injury [17, 20], and three developed CSW between 2 weeks and 2 months after injury [19, 21, 24]. In the adult cases, one patient’s CSW occurred approximately 1 week post-injury [16] and the other adult case did not specify time between injury and CSW, though it was noted that CSW occurred within 24 h after ending their pentobarbital infusion [15].

### Biochemical changes

Table 1 summarizes select hormones reported in the included studies. Three case studies reported increased levels of ANP [20–22], while one case study and one prospective study reported no increase in ANP amongst patients who developed CSW [23, 24]. Lu et al. described two cases of CSW with elevated BNP levels [16]; however, two prospective studies [12, 23] and one additional case report [24] found no correlation between BNP and serum sodium level. ADH was normal [12, 23] or decreased [14, 21] in patients with CSW after TBI. Donati-Genet et al. described a CSW case in which the ADH was initially increased, but rapidly normalized after volume and sodium supplementation [20]. Aldosterone levels were normal [12] or decreased [20–22], cortisol and ACTH were within a normal range [19–21, 24], and plasma renin activity was decreased [20–22] in patients who developed CSW after TBI.

**Table 1:** Summary of select hormones in patients who developed cerebral salt wasting after traumatic brain injury

Author	ANP	BNP	ADH	Aldosterone	Cortisol	ACTH	Plasma renin activity
Cohort Studies							
Costa [12]		Normal	Normal	Normal			
Zhang [23]	Normal or ↓	Normal or ↓	Normal				
Vingerhoets [14]			↓				
Case Reports							
Simsek [19]					Normal		
Askar [24]	Normal	Normal			Normal		
Lu [16]		↑					
Donati-Genet [20]	↑		↑^a^	↓	Normal	Normal	↓
Kappy [21]	↑		↓	↓	Normal		↓
Ganong [22]	↑			↓			↓

*ANP* atrial natriuretic peptide, *BNP* Brain natriuretic peptide, *ADH* Antidiuretic hormone, *ACTH* Adrenocorticotropic hormone

^a^ rapidly normalized after volume and sodium supplementation

### Outcomes

All of the case reports provided information on clinical outcomes; of those, one patient (10 %) died [16] and the remaining nine improved. Prior to their CSW diagnosis, two patients developed seizures [19, 20]. The cohort studies often described the incidence, etiology, and biochemical changes observed in patients, but few reported patient outcomes. Lohani et al. reported no significant difference in hospital length of stay (LOS, *p* = 0.83) or Glasgow Outcome Scale (GOS, *p* = 0.55) at discharge for hyponatremic versus non-hyponatremic patients [11]. Moro et al. found hyponatremia was associated with longer LOS (*p* < .001) and a bad outcome (severe disability, vegetative state, or death on GOS) 1 month after hospital discharge (*p* = 0.02) [13]. Neither study reported outcomes specifically for CSW patients.

## Discussion

This review focuses on the CSW literature within the TBI population. We found much uncertainty surrounding CSW. In particular, the reported incidence of CSW is wide. Small study size and varying populations, study inclusion, and CSW diagnostic criteria may contribute to the wide range in incidence. Hyponatremia is typically noted before CSW is suspected. Among cohort studies in this review, criteria to diagnose CSW or SIADH included natremia levels of 130 [11, 12], 135 [14, 23], 136 [13], and 137 mmol/L [18]. To confidently diagnose CSW, regardless of etiology, we believe the following criteria must be met:

1. Brain Pathology: new in onset or severity and defined by neurologic exam, neuro-imaging, cerebral spinal fluid examination, laboratory testing, or electroencephalogram. Vacillation in the neurologic assessment should track with indices of sodium and water balance.
2. Hyponatremia: validated by at least one simultaneously low serum osmolality.
3. Hypovolemia: clinical assessment of intravascular volume is difficult to accomplish, yet needs to be assessed.
4. Urinary salt loss: Two caveats apply; first, a high urine sodium does not prove net salt loss, which can only be calculated by knowing the volume and sodium concentration of urine during an interval of time and comparing it to sodium intake for that same period. Second, a salt loss in excess of salt intake would not be “wastage” if the patient had prior extracellular fluid expansion that induced a “corrective” natriuresis.

Implicit in the diagnosis of CSW is the reasonable certainty that other known causes of renal salt loss have been excluded. The following is a far from exhaustive list of clinically important factors to consider when determining if a patient is experiencing CSW:

1. Fluid overload from initial resuscitation.
2. Episodic catecholamine surges induced by central nervous system injury that can increase blood pressure, cause pressure natriuresis, and cause vasoconstriction of capacitance vessels [27].
3. When co-existent cervical spinal cord injury with tetraplegia exists, the position of the patient when tested is critical. Greater natriuresis and aquaresis occur when supine and are attenuated when upright [28].
4. Adrenal insufficiency must be excluded, with special emphasis on mineralocorticoid insufficiency.
5. Intrinsic renal tubular injury must be excluded. To implicate acute kidney injury as a cause of urinary salt wastage requires tests of greater sensitivity and specificity than blood urea nitrogen or creatinine, which reflect glomerular rather than renal tubular function. The renal assessment should include fractional excretions of sodium, chloride, phosphate, and urate and could include tests of tubular proteinuria and aminoaciduria.
6. An ever-increasing number of medications will alter renal tubular function, including diuretics, osmotic agents, intravenous radiocontrast, topiramate, aminoglycosides, caffeine, theophylline, and guaifenisin.

Adding to the ambiguity in diagnosing CSW is the uncertainty surrounding the mechanisms causing CSW. It has been suggested CSW takes a different course in adults compared to children, with CSW being a delayed phenomenon in adults [17, 29] and developing earlier in children [17]. Our review resulted in few adult cases making it difficult to compare the timing from injury to CSW in adults; however, within children we found varied times to onset. The timing from injury to onset of CSW may be indicative of more than one pathophysiology causing CSW after TBI [13], with pathways influenced by the type and location of injury, as well as physiological differences between adults and children.

The leading theory purports increased levels of natriuretic peptides and changes in the sympathetic nervous system contribute to the development of CSW [4, 7, 29]. ANP, BNP, C type natriuretic peptide (CNP), and dendroaspis natriuretic peptide (DNP) have been associated with CSW [7]. These hormones inhibit sympathetic outflow and the production of vasoconstrictor peptides, inhibit the renin-angiotensin-aldosterone system, and act as vasodilators [30]. After intracranial insult the natriuretic peptides may increase because of atrial stretch and increased ventricular load from surges in sympathetic outflow [31], direct damage to the brain structures housing BNP involuntarily releasing the peptide into circulation [7] and, the hypothalamus, which may generate and release ANP and BNP as a protective mechanism against rising intracranial pressure and potential unfavorable outcomes [32].

While elevated levels of ANP [33, 34] and BNP [31, 32, 35] have been reported in aSAH patients, we found neither ANP nor BNP unfailingly increased in TBI patients with CSW [20–24]. Interestingly, head CT scans revealed bleeds in the two cases with elevated BNP. Though reported to be smaller bleeds than typically seen with aSAH, it cannot be ruled out that the hemorrhage was responsible for increased levels of BNP [16]. In the studies with elevated ANP [20–22], aldosterone was decreased, which may reflect ANP inhibiting the renin-angiotensin-aldosterone system [30].

Still, additional mechanisms causing CSW warrant study. Kojima et al. suggested the cause of CSW is neither ANP, BNP, nor ADH, but rather a novel mechanism or endogenous compound [36].

### Limitations

Our review has several limitations. Few studies addressed CSW in the TBI population and those that do are of relatively low quality. We found just fifteen publications eligible for inclusion in our study, nine of which were case reports. Patient populations were heterogeneous and diagnostic criteria used to define CSW varied to such a wide extent that we found it necessary to take at face value what others defined as CSW. Very importantly, little information was reported on TBI patient outcomes after developing CSW.

## Conclusions

Although the mechanisms are poorly understood, it is recognized that distinguishing CSW from SIADH and other etiologies of hyponatremia is important when treating patients with brain injuries. We found the incidence and timing of CSW varies widely in TBI patients, from 0.8–34.6 % and develops within days to two months post-injury. While theories exist, the mechanisms of CSW are unclear and the association of natriuretic peptides is inconsistently reported. The TBI population is complicated as TBIs arise from various and diverse initial insults, occur in patients of all ages, and may result in contusions, hemorrhages, or edema. The confounding factors highlighted in this review, in combination with the disparate incidence, emphasize the exigency for strict and consistent diagnostic criteria for CSW, not only to be used clinically, but also when presenting research pertaining to CSW. Only then can we gain a better understanding of CSW, the most elusive of the causes of hyponatremia.

## Additional files

Additional file 1: Table S1. Summary of Cohort Studies examining cerebral salt wasting after traumatic brain injury. (DOCX 19 kb) Additional file 2: Table S2. Summary of Case Reports examining cerebral salt wasting after traumatic brain injury. (DOCX 19 kb)

## Abbreviations

AAST: American Association for the Surgery of Trauma
ACTH: Adrenocorticotropic hormone
ADH: Antidiuretic hormone
ANP: Atrial natriuretic peptide
aSAH: Aneurysmal subarachnoid hemorrhage
BNP: Brain natriuretic peptide
CNP: C type natriuretic peptide
CSW: Cerebral salt wasting
CT: Computed tomography
DNP: Dendroaspis natriuretic peptide
GCS: Glasgow coma score
GOS: Glasgow outcome score
LOS: Length of stay
PRISMA: Preferred reporting items for systematic reviews and meta-analyses
SIADH: Syndrome of inappropriate antidiuretic hormone
TBI: Traumatic brain injury
tSAH: Traumatic subarachnoid hemorrhage
