# Supplementary material for: Cerebral salt wasting after traumatic brain injury: a review of the literature
Source: Scand J Trauma Resusc Emerg Med. 2015 Nov 11;23:98. doi: 10.1186/s13049-015-0180-5 (PMC4642664; doi:10.1186/s13049-015-0180-5)
Supplement: Additional file 1: Table S1. — Summary of Cohort Studies examining cerebral salt wasting after traumatic brain injury. (DOCX 19 kb) [file 13049_2015_180_MOESM1_ESM.docx]

| Additional File Table 1. Summary of **Cohort Studies** examining cerebral salt wasting after traumatic brain injury | | | | | | | |
| --- | --- | --- | --- | --- | --- | --- | --- |
| **Author**  **(Year)** | **Country** | **Study Design and**  **Inclusion Criteria** | **Exclusion Criteria** | **Age**^a^ | **Percent Male** | **Head CT / MRI Findings** | **CSW Incidence**  **(cases/n)** |
| **Lohani** [11]  (2011) | Nepal | **Prospective**  ≥20 years; Admitted within 3 days after TBI | Renal, thyroid, adrenal disease prior to TBI; Spinal cord injury | 37.4±15.3  (19-70) | 79% | Intraparenchymal lesion (67%);  Epidural lesion (24%); Subdural lesion (12%); tSAH (15%); Pneumocephalus (9%) | 9.1%  (3/33) |
| **Zhang** [23]  (2010) | China | **Prospective**  Hospitalized <24 hours after craniocerebral injury, confirmed by CT | Other severe injuries/ shock; History of alcohol use before injury, hypertension/ other heart disease; Abnormal organ function; Glucocorticoid hormone after injury | 27.8  (4-60) | 75% | -- | 29.4%  (20/68) |
| **Costa [**12]  (2009) | Brazil | **Prospective**  Admitted to trauma ICU for brain injury and GCS <9 | Cardiopathy, acute/chronic renal failure, brain death signs at admission; Trauma ICU admission >1 day after event; Glucocorticoid & diuretics during study period; Trauma ICU stay <10 days | 29.1±9  (18-54) | 85% | Brain Swelling (65%); Intracranial Bleed (10%); Brain Contusion (31%) | 34.6%  (9/26) |
| **Moro** [13]  (2007) | Japan | **Retrospective**  Admitted with TBI | Admitted for observation with no abnormal lesions; Spinal cord injury; Posttraumatic vomiting | 49.3  (0-92) | 66% | Chronic SDH (30%); Concussion (21%); Skull fracture (16%); Cerebral contusion (16%); Acute SDH (8%); Acute epidural hematoma (5%); tSAH (3%) | 4.4%  (13/298) |
| **Einaudi**^b^ [18]  (2006) | Italy | **Prospective**  Consecutive TBI patients admitted to neurosurgical department or ICU (pediatric hospital) |  | 9.1  (0.25-15.5) | 77% | -- | 10%  (3/30) |
| **Vingerhoets** [14]  (1988) | Switzer-  land | **Prospective**  Neurosurgical department admit for severe head injury |  | -- | -- | -- | 0.8%  (2/256) |

^a^mean ± standard deviation (range); ^b^Excluded retrospective portion of the study

-- = Not Reported; ATV=all-terrain vehicle; CSW=cerebral salt wasting; CT=computed tomography; GCS=Glasgow Coma Scale; IC=intracerebral; ICU=intensive care unit MRI=magnetic resonance imaging; MVA=motor vehicle accident; SDH=subdural hematoma; TBI=traumatic brain injury; tSAH=traumatic subarachnoid hemorrhage
