# Supplementary material for: Cerebral salt wasting after traumatic brain injury: a review of the literature
Source: Scand J Trauma Resusc Emerg Med. 2015 Nov 11;23:98. doi: 10.1186/s13049-015-0180-5 (PMC4642664; doi:10.1186/s13049-015-0180-5)
Supplement: Additional file 2: Table S2. — Summary of Case Reports examining cerebral salt wasting after traumatic brain injury. (DOCX 19 kb) [file 13049_2015_180_MOESM2_ESM.docx]

| Additional File Table 2. Summary of **Case Reports** examining cerebral salt wasting after traumatic brain injury | | | | | |
| --- | --- | --- | --- | --- | --- |
| **Author**  **(Year)** | **Country** | **Brief Description of Case** | **Head CT / MRI Findings** | **Timing to CSW Development** | **Patient**  **Outcome** |
| **Kontogiorgi** [15]  (2011) | Greece | **22 year old male**: TBI treated with therapeutic barbiturate coma | SAH; cerebral edema | <24 hours after ending pentobarbital infusion | Improved |
| **Simsek** [19]  (2008) | Turkey | **6 month old female**: Closed head & cervicothoracic trauma due to MVA | 8mm benign congenital subdural collection over frontotemporal lobes; No IC bleeding or edema; Day 5 MRI: Edema & SDH between C6 and T4 | 1 month post-MVA | Improved |
| **Lu**^a^ [16]  (2008) | United States | **52 year old male**: Assault to the head | Large right SDH with 8mm midline shift; Extensive right frontal contusions; IVH; Right uncal herniation | Approximately 1 week after assault | Died |
| **Askar** [24]  (2007) | Saudi Arabia | **17 year old male**: Closed head trauma & multiple injuries to face, chest, and pelvis due to MVA | No evidence of SDH, SAH, IC bleeding or cerebral edema | 15 days post-MVA | Improved |
| **Steelman** [25]  (2006) | United States | **9 year old male**: Laceration to chin & closed head trauma due to fall from 20-foot embankment while riding ATV | No evidence subdural / epidural hematoma, SAH or intraparenchymal bleeding; No midline shifts or cerebral edema | 2 days after accident | Improved |
| **Berkenbosch**^b^ [17]  (2002) | United States | Patient 1 - **15 year old male**: Severe closed head injury from cycling accident  Patient 2 - **6 year old male**: Severe closed head injury | Patient 1: right-sided frontal contusion  Patient 2: 1.5 cm left frontoparietal contusion, marked diffuse cerebral edema | Patient 1: 2 days post-accident  Patient 2: 6 days post-injury | Both  Improved |
| **Donati-Genet** [20]  (2001) | Switzer-land | **4 year old male**: Closed head injury, multiple bone fractures, chest trauma with bilateral lung contusions & pneumothoraces after fall from 5th floor of building | Small occipital epidural hematoma  Day 5 CT after seizure: diffuse cerebral edema & small cerebellar hemorrhage | Day 5 post-accident | Improved |
| **Kappy** [21]  (1996) | United States | **6 month old male**: MVA with normal initial evaluation. Over next 2 months, vomiting & increasing head circumference | 2 months post-accident bilateral subdural fluid accumulation | 2 months post-MVA | Improved |
| **Ganong** [22]  (1993) | United States | **5 year old male**: Closed head injury due to MVA | Not Reported | Approximately 2 days after injury | Improved |

^a^Excluded second case due to a pre-existing history of alcohol abuse; ^b^Excluded case of CSW not due to traumatic brain injury

ATV=all-terrain vehicle; CSW=cerebral salt wasting; CT=computed tomography; IC=intracerebral; IVH=intraventricular hemorrhage; MRI=magnetic resonance imaging; MVA=motor vehicle accident; SAH=subarachnoid hemorrhage; SDH=subdural hematoma
